# Multi-shell gold nanoparticles functionalized with methotrexate: a novel nanotherapeutic approach for improved antitumoral and antioxidant activity and enhanced biocompatibility

**DOI:** 10.1080/10717544.2024.2388624

**Published:** 2024-08-17

**Authors:** Denisse-Iulia Bostiog, Natalia Simionescu, Adina Coroaba, Ioana C. Marinas, Mariana C. Chifiriuc, Gratiela Gradisteanu Pircalabioru, Stelian S. Maier, Mariana Pinteala

**Affiliations:** a Centre of Advanced Research in Bionanoconjugates and Biopolymers, “Petru Poni” Institute of Macromolecular Chemistry, Iasi, Romania; b Department of Microbiology and Immunology, Research Institute of the University of Bucharest-ICUB, Bucharest, Romania; c Polymer Research Center, “Gheorghe Asachi” Technical University of Iasi, Iasi, Romania

**Keywords:** Gold nanoparticles, multi-shell nanoparticles, methotrexate, drug delivery, biocompatibility, antitumoral activity, antioxidant activity

## Abstract

Methotrexate (MTX) is a folic acid antagonist routinely used in cancer treatment, characterized by poor water solubility and low skin permeability. These issues could be mitigated by using drug delivery systems, such as functionalized gold nanoparticles (AuNPs), known for their versatility and unique properties. This study aimed to develop multi-shell AuNPs functionalized with MTX for the improvement of MTX antitumoral, antioxidant, and biocompatibility features. Stable phosphine-coated AuNPs were synthesized and functionalized with tailored polyethylene glycol (PEG) and short-branched polyethyleneimine (PEI) moieties, followed by MTX covalent binding. Physicochemical characterization by UV–vis and Fourier-transform infrared spectroscopy (FTIR) spectroscopy, dynamic light scattering (DLS), scanning transmission electron microscopy (STEM), and X-ray photoelectron spectroscopy (XPS) confirmed the synthesis at each step. The antioxidant activity of functionalized AuNPs was determined using DPPH radical scavenging assay, ferric ions’ reducing antioxidant power (FRAP), and cupric reducing antioxidant capacity (CUPRAC) assays. Biocompatibility and cytotoxicity were assessed using MTT and LDH assays on HaCaT human keratinocytes and CAL27 squamous cell carcinoma. MTX functionalized AuNPs demonstrated enhanced antioxidant activity and a pronounced cytotoxic effect on the tumoral cells compared to their individual components, highlighting their potential for improving cancer therapy.

## 1. Introduction

Cancer has remained a prominent cause of morbidity and mortality over the past several decades and efforts to develop new drugs, to enhance the efficacy of existing ones, and to achieve the desideratum of targeted therapies, are equally crucial (Yang et al., 2020; Khan & Torchilin, 2022). Furthermore, cancer-related skin conditions (rashes, pruritus, peeling, xerosis, sores, pressure ulcers, pain, malignant wounds, etc.) are common, ranging from uncomfortable to serious reactions. They represent manifestations of different types of cancer (breast, head and neck, primary skin, genital, gastrointestinal cancers, and lymphoma) or they can occur as side effects of oncologic treatments (Stolnicu et al., 2017; Mihai et al., 2021). Another topical complication is represented by malignant wounds which can be the result of primary skin cancer, local extension from primary tumors, or metastasis to the skin, and represent the invasion of cancer cells into the epithelium and dermal blood and lymph vessels, creating a non-healing wound (O’Brien, 2012). They are characterized by tissue death and necrosis, inflammation, infection, bleeding, odor, and drainage (O’Brien, 2012). Despite the psychological aspects and important challenges such as pain, exudate, bleeding, pruritus, malodor, impaired mobility, infection, nausea, and anorexia, there is no standardized treatment for malignant wounds (Qiu & DelVecchio Good, 2021). Therefore, the therapeutic management of these skin conditions represents an important part of supportive cancer care, combining local (sunscreen, moisturizing creams, or ointments supplemented with different active substances) and systemic agents (antibiotics, corticosteroids, antihistamines, etc.).

One well-known adjuvant for the treatment of various cancers, including leukemia, lymphoma, head and neck cancer, and breast tumors (Yang et al., 2020), is methotrexate (MTX), a folate antagonist. MTX has poor water solubility and low permeability, necessitating the administration of higher doses over time, which decreases its bioavailability for longer periods. To overcome these problems, new strategies should be used for efficient and longer administration of MTX, such as the use of functionalized nanostructures like gold nanoparticles (AuNPs), which show promising potential for personalized cancer therapy, by directing the chemotherapeutic drugs to the intended target cells and maintaining sufficient intracellular concentrations to inhibit their growth and functions (Álvarez-González et al., 2020; Khodashenas et al., 2021; Pasparakis, 2022; Xu et al., 2022). Other nano-approaches have been used to enhance MTX properties and delivery to tumors, such as iron oxide magnetic nanoparticles (Nosrati et al., 2018), poly(lactic-co-glycolic acid) nanoparticles (Vakilinezhad et al., 2019), chitosan/TiO_2_ nanoparticles (Al-Nemrawi et al., 2022), or polymeric microcapsules (Al-Nemrawi et al., 2018).

In the 1850s, Michael Faraday (Faraday, 1857) first created and named ‘divided gold metal’, which is now known as colloidal gold. In a chemical bottom-up approach, AuNPs with different shapes can be synthesized. Later, Bansal et al. explained that the colorful appearance of Faraday’s gold solution is caused by the absorption and scattering of light by the nano-sized particles (Bansal et al., 2020). The AuNPs’ optical and electronic properties depend on their shape and size, as a result of the surface plasmon resonance (SPR) effect (Trouiller et al., 2015; Sharifi et al., 2019).

The unique optical properties of AuNPs have made them valuable in biomedical applications such as photothermal therapy, where they can be coated with selective moieties that target cancer cells (Khodashenas et al., 2021), or in the field of bioimaging, where AuNPs can be used as probes to visualize cellular compartments and track their uptake, specificity, and location within cells, based on their distinct color-changing ability (Nejati et al., 2022). Furthermore, AuNPs exhibit special physicochemical characteristics, such as chemical inertness, ease of surface functionalization, good biocompatibility, and simple ligand exchange functionalization, which improves their complexing capacity for medicinal compounds (Tran et al., 2013; Wang et al., 2016).

Gold nanoparticles make excellent drug carriers due to their small size, high penetrability, and stability. Various techniques such as physical adsorption, ionic bonding, and covalent bonding can be used to attach drugs to nanoparticles, ensuring that the drugs remain within the cells for sufficient time to exert their therapeutic effects (Pasparakis, 2022; Xu et al., 2022).

In this study, we propose a novel formulation to enhance the efficacy (antitumoral and antioxidant properties) and safety of MTX, by using two distinct systems of AuNPs as carriers, to create multi-shell AuNPs functionalized with MTX. By binding MTX to AuNPs, it can potentially accumulate better within the tumor cells for a longer duration, leading to improved pharmacokinetic behavior and a stronger therapeutic effect (Fratoddi et al., 2019).

The synthesis process involved several steps, starting with the functionalization of small and stable phosphine-coated AuNPs using tailored oligomers of poly(ethylene glycol) (PEG) for covalent attachment. The second shell was then created by coupling short-branched poly(ethylene-imine) (PEI) moieties, followed by the covalent binding of MTX. The functionalized AuNPs were subjected to a comprehensive analysis using various techniques, including ultraviolet–visible (UV–vis) spectroscopy, Fourier-transform infrared spectroscopy (FTIR), dynamic light scattering (DLS), zeta potential, scanning transmission electron microscopy (STEM), and X-ray photoelectron spectroscopy (XPS). The purpose of this evaluation was to determine the nature and degree of functionalization at each step of the preparation process. The antioxidant efficiency of functionalized AuNPs was determined using DPPH radical scavenging assay, ferric ions’ reducing antioxidant power (FRAP), and cupric reducing antioxidant capacity (CUPRAC) assays. Finally, the nanoparticles’ biocompatibility and cytotoxicity were evaluated *in vitro* using LDH and MTT assays on normal keratinocytes and CAL27 squamous cell carcinoma, respectively.

## 2. Materials and methods

### 2.1. Materials

Gold (III) chloride trihydrate (HAuCl_4_·3H_2_O; ≥99.9%), bis(p-sulfonatophenyl) phenyl phosphine dihydrate dipotassium salt (C_18_H_13_K_2_O_6_PS_2_·2H_2_O; 97%), PEG (HS-PEG_1000_-COOH), polyethyleneimine (PEI) branched (H(NHCH_2_CH_2_)_800_NH_2_), methanol (CH_3_OH; 99.8%), N-ethyl-N′-(3-dimethylaminopropyl) carbodiimide hydrochloride (EDC, C_8_H_17_N_3_·HCl; ≥98.0%), N-hydroxy succinimide (NHS, C_4_H_5_NO_3_; 98%), N,N-diisopropylethylamine (DIEA, [(CH_3_)_2_CH]_2_NC_2_H_5_; ≥99%), MTX (C_20_H_22_N_8_O_5_), dimethyl sulfoxide (DMSO, ≥99.9%) were purchased from Sigma-Aldrich (St. Louis, MO), while tri-sodium citrate dihydrate (C_6_H_5_Na_3_O_7_·2H_2_O; 99%) was purchased from Carl Roth (Karlsruhe, Germany), thiol PEG amine (HS-PEG_2000_-NH_2_HCl, MW 2000) was purchased from JenKem Technology (Beijing, China), and sodium chloride (NaCl) was purchased from Chimopar (Bucharest, Romania). Unless otherwise specified, all materials were used as supplied, with no further purification.

### 2.2. Synthesis of gold nanoparticles

AuNPs were successfully produced by adapting the Turkevich approach (Polte et al., 2010; Wuithschick et al., 2015). First, gold (III) chloride (12.5 mg) was added to 100 mL of MQ water in a 250 mL flask. The flask was then placed into a preheated oil bath at 110 °C, under vigorous stirring (700 rpm). Once the gold salt was dissolved and the temperature reached approximately 80–85 °C, a heated solution of sodium citrate (50 mg, in 50 mL of MQ water) was added to the flask (the whole amount as soon as possible).

The solution was left in the oil bath under stirring and heating for 15 minutes. Afterwards, the flask was removed from the oil bath and left for one hour under continuous stirring at room temperature, resulting in a ruby-red solution.

### 2.3. Phosphine coating of AuNPs (AuNPs-p)

A modified previously published method was used to create phosphine-coated AuNPs (Ursu et al., 2017). Phosphine coating stabilizes AuNPs in high electrolyte concentrations and higher particle density by increasing the negative charge on their surface (Ding et al., 2010). The method involved adding 2 mg of phosphine to 15 mL of AuNPs under light-protected conditions and allowing them to stir gently for 24 hours. NaCl (∼2 mg) was added until the color changed from red to blue. The sample was then centrifuged for 10 minutes at 10,000 rpm, and the supernatant was removed. To resuspend the particles, 0.3 mL of a 2.5 mM phosphine solution was added, followed by the addition of 0.5 mL of methanol. The solution turned black after the addition of methanol. Following centrifugation at 10,000 rpm for 10 minutes, the supernatant was carefully removed, and the particles were resuspended in 0.2 mL of phosphine solution. The resulting mixture was then transferred to an Eppendorf tube and diligently stored at 4 °C, shielded from light exposure.

### 2.4. PEGylation of AuNPs-phosphine (AuNPs–PEG–NH_2_ and AuNPs–PEG–COOH)

In order to facilitate the subsequent functionalization with MTX, two intermediate samples were prepared by PEG-coating of nanoparticles, AuNPs–PEG–NH_2_ and AuNPs–PEG–COOH, respectively. To synthesize AuNPs–PEG–NH_2_, 19.6 µL of HS-PEG_2000_-NH_2_ (1 mg/mL) was added to 600 µL of AuNPs-p. In order to prepare AuNPs–PEG–COOH, the PEG-coated nanoparticles for use in the next synthesis step which involves the PEI coupling, 9.45 µL of HS-PEG_1000_-COOH (1 mg/mL) was added to 600 µL of AuNPs-p. Both samples were incubated at room temperature under light-protected conditions and continuously stirred in a thermoshaker for 48 hours. After incubation, the samples were filtered using Amicon Ultra centrifugal filters (4 mL, 10K, Merck KGaA, Darmstadt, Germany), and resuspended in 500 µL of ultra-pure water. The samples were then stored at 4 °C, in a light-protected environment.

### 2.5. Short-branched PEI coupling (AuNPs–PEG–PEI)

To activate the carboxylic group for additional PEI (PEI_800_) coupling, a reaction using N-ethyl-N′-(3-dimethylaminopropyl) carbodiimide hydrochloride (EDC), NHS, and DIEA was conducted (Wang et al., 2016). Specifically, 25 µL of EDC (1.5 M), 25 µL of NHS (0.075 M), and 3 µL of DIEA were added to 600 µL of AuNPs–PEG–COOH and left to stir for three hours at 550 rpm. An excess of PEI (15 mg, 0.018 mmol) was then added and stirred for 72 hours. The resulting solution was filtered using Amicon Ultra centrifugal filters (4 mL, 100K, Merck KGaA, Darmstadt, Germany) and resuspended in 500 µL of ultra-pure water. The sample was stored at 4 °C, in the dark.

### 2.6. Preparation of MTX-conjugated gold nanoparticles (AuNPs–PEG–MTX and AuNPs–PEG–PEI–MTX)

The coupling chemistry approach was applied to modify the AuNPs using the carboxyl activators DIEA and EDC in DMSO, targeting the carboxyl groups in the MTX structure. Specifically, 0.0025 g EDC, 18 µL DIEA, and 500 µL DMSO were added to 0.005 g MTX, and the resulting mixture was stirred for 24 hours to activate the carboxyl groups. Following that, 60 µL of the prepared MTX solution was mixed with 600 µL of AuNPs–PEG–NH_2_, to produce the first AuNPs–PEG–MTX nano-entity. An additional 60 µL of the MTX solution was added to 600 µL of AuNPs–PEG–PEI to generate the second AuNPs–PEG–PEI–MTX nano-entity. The mixtures were stirred for an additional 24 hours, in the dark. After that, the suspensions were purified using Amicon Ultra centrifugal filters (0.5 mL, 10K, Merck KGaA, Darmstadt, Germany), to obtain the MTX-functionalized AuNPs.

### 2.7. Characterization

The UV–vis spectroscopy measurements were carried out using a Lambda 35 instrument (Perkin Elmer, Waltham, MA). To ensure equivalent sample quantities, 2 mL of each sample was used, and absorption spectra were measured in the 300–700 nm range using a slit width of 1 nm, a scan speed of 480 nm/min, and a data interval of 1 nm. Quartz cuvettes with a 1 cm path length were used to measure the spectra of the samples at room temperature. The spectra for the nanoparticles were recorded after diluting the samples in a ratio of 1:20.

A Bruker Vertex instrument model 70 (Billerica, MA) was used to obtain the FTIR spectra in transmission mode. The samples were made by depositing a nanoparticle suspension over potassium bromide, which were subsequently dried (with the help of a UV lamp) before the spectra were taken. The resolution of the spectra, which spanned from 4000 to 400 cm^−1^, was 2 cm^−1^.

Dynamic light scattering and zeta potential were used to measure the hydrodynamic diameter and zeta potential of the nanoparticles using a Delsa Nano C Submicron Particle Size Analyzer with Flow Cell Module (from Beckman Coulter, Brea, CA).

Various G4 UC scanning electron microscope equipped with a STEM 3+ detector (Thermo Fisher Scientific, Waltham, MA) operating in STEM mode at 30 kV was used to examine the morphology of the samples (Bright-Field Mode). Samples (10–20 μL) were fixed on 24 carbon-coated copper grids with 300-mesh size and air-dried. A size distribution histogram was plotted after manually measuring the sizes of roughly 100 particles for each sample.

An Axis NOVA instrument (Kratos Analytical, Manchester, UK) was used to acquire the XPS data. The X-ray source used was AlK with an energy of 1486.6 eV. The instrument was operated at 300 W (equivalent to a current of 20 mA and a voltage of 15 kV). The pressure during the measurements ranged from 10^−8^ to 10^−9^ Torr. High-resolution spectra were acquired for each element of interest by averaging five scans that were recorded using a pass energy of 20 eV and a step size of 0.1 eV. C 1s peak, normalized at 285 eV, was the reference value for all binding energies. The CasaXPS software, version 2.3.25, was used to perform the fitting procedure.

### 2.8. Antioxidant activity

#### 2.8.1. DPPH radical scavenging assay

DPPH assay was performed according to the protocol described by Madhu et al. (2013), with slight changes. Specifically, the reaction mixture was composed of 50 µL of sample/standard and 50 µL of 0.3 mM DPPH radical methanolic solution. The absorbance was measured at *λ* = 517 nm after 20 min of incubation in the dark and 5 min centrifugation at 7000 rpm, using a Multiskan FC UV-VIS spectrophotometer (Thermo Fisher Scientific, Waltham, MA). The concentrations used for the Trolox calibration curve were in the range of 5–80 µM (*R*^2^ = 0.9975). Sodium lignosulfonate (NaLS) with a concentration of 1 mg/mL was used as a standard antioxidant solution (from Carl Roth, Karlsruhe, Germany).

#### 2.8.2. Ferric ions’ reducing antioxidant power assay

The FRAP assay was carried out according to the protocol outlined by Thaipong et al. (2006). The reaction mixture was composed of 475 µL of FRAP reagent (prepared according to Multescu et al., 2022: 300 mM acetate buffer (3.1 g C_2_H_3_NaO_2_·3H_2_O and 16 mL C_2_H_4_O_2_), pH 3.6, 10 mM 2,4,6-tripyridyl-s-triazine (TPTZ) solution in 40 mM HCl, and 20 mM FeCl_3_·6H_2_O solution; the fresh working solution was prepared by mixing 25 mL acetate buffer, 2.5 mL TPTZ solution, and 2.5 mL FeCl_3_·6H_2_O solution, and then incubated at 37 °C before use) and 25 µL of sample/standard. The absorbance was measured at *λ* = 593 nm after 20 min of incubation in the dark at 37 °C and 5 min centrifugation at 7000 rpm, using a Multiskan FC UV-VIS spectrophotometer (Thermo Fisher Scientific, Waltham, MA). A stock solution of Trolox with a concentration of 1 mM was used to plot the calibration curve. The concentration of Trolox in the range of 30–250 µM was employed for this purpose, resulting in an *R*^2^ value of 0.9978, and NaLS (from Carl Roth, Karlsruhe, Germany) solution of 1 mg/mL was used as a standard antioxidant.

#### 2.8.3. Cupric reducing antioxidant capacity assay

The CUPRAC assay was performed according to the protocol described by Celik et al. (2010). Specifically, 60 μL of sample/standard solutions of varying concentrations were mixed with 50 μL of CuCl_2_ (10 mM), 50 μL of neocuproine (7.5 mM), and 50 μL of 1 M ammonium acetate buffer at a pH of 7. After 30 min of incubation, the absorbance was measured at 450 nm. The concentrations used for the Trolox calibration curve were in the range of 0.125–1.5 mM (*R*^2^ = 0.9983), and NaLS (from Carl Roth, Karlsruhe, Germany) solution of 1 mg/mL was used as a standard antioxidant.

### 2.9. Cell viability assessment

The biocompatibility and cytotoxicity of MTX-functionalized AuNPs were tested on HaCaT human keratinocytes (purchased from Cell Line Services (CLS), Eppelheim, Germany, product number: 300493, RRID: CVCL_0038) and CAL27 squamous cell carcinoma from the oral cavity (purchased from American Type Culture Collection – ATCC, Manassas, VA, catalogue no. CRL-2095, RRID: CVCL_1107). The cells were grown in DMEM medium (Dulbecco’s modified Eagle medium) supplemented with 1% Pen/Strep (penicillin/streptomycin solution, 50 µg/mL) and 10% fetal bovine serum (all purchased from Sigma-Aldrich, St. Louis, MO) for 24 hours at 37 °C, 95% humidity with 5% CO_2_. Cells were washed with saline (Sigma-Aldrich, St. Louis, MO), trypsinized (trypsin–EDTA 0.25%, Thermo Fisher Scientific, Waltham, MA), and counted using a hemocytometer and Trypan Blue. The nanoparticles were co-cultured with the cells (a seeding density of 5 × 10^5^ cells/well) for 24 hours (37 °C, 95% humidity, 5% CO_2_). Control cells were incubated with a complete cell culture medium.

#### 2.9.1. MTT assay

The MTT assay (Vybrant^®^ MTT Cell Proliferation Assay Kit, cat. no. V-13154, Thermo Fisher Scientific, Waltham, MA) was used to assess cell viability in the presence of nanoparticles. The cells were incubated with MTT reagent at 37 °C, 95% humidity with 5% CO_2_, then isopropanol was used to solubilize the formazan crystals, and the optical densities were recorded at 550 nm using a Multiskan FC UV–VIS spectrophotometer (Thermo Fisher Scientific, Waltham, MA).

#### 2.9.2. LDH release assay

The cytotoxicity of the nanoparticles was evaluated using the LDH cytotoxicity detection kit from Roche Holding AG, cat. no. 11644793001 (Basel, Switzerland). The LDH activity was measured in the collected cell culture supernatant according to the manufacturer’s guidelines through a Multiskan FC UV-Vis spectrophotometer (Thermo Fisher Scientific, Waltham, MA) at *λ* = 490 nm, with a reference wavelength of *λ* = 600 nm.

#### 2.9.3. Live/dead assay

Viability of the cells was analyzed using the Live/Dead assay (Thermo Scientific, Waltham, MA). Imaging was performed at *λ* = 494/517 (live cells) and at *λ* = 517/617 (dead cells) using a fluorescence microscope (Zeiss AxioScope equipped with an Axiocam 506 mono camera, Oberkochen, Germany).

### 2.10. Statistical analysis

Data obtained in triplicate were expressed as means ± standard deviation. GraphPad Prism v9 (GraphPad Software, La Jolla, CA) was used for the statistical analysis. The data were subjected to one-way or two-way analysis of variance (ANOVA), with no correction for multiple comparisons (performed by Fisher’s LSD test to determine which NPs behave significantly differently) in the case of antioxidant activity. The cell viability findings were evaluated by ordinary two-way ANOVA using a two-stage linear step-up procedure of Benjamini, Krieger, and Yekutieli, with a single pooled variance method, for estimating false discovery rate and to accordingly adjust *p* values for pairwise comparisons. The level of statistical significance was set at *p* < .05.

## 3. Results and discussion

In order to increase the stability, concentration, and charge density of AuNPs, bis(p-sulfonate phenyl)phenylphosphine dihydrate dipotassium salt was added (Ursu et al., 2017) to the solution of AuNPs obtained by the Turkevich method. This procedure allowed the separation of nanoparticles by centrifugation and then their resuspension in high concentration.

Next, two nano-entities (AuNPs–PEG–NH_2_ and AuNPs–PEG–COOH) were generated through the covalent attachment, involving the thiol function, of two well-defined PEG derivatives (HS-PEG_2000_-NH_2_ and HS-PEG_1000_-COOH, respectively) to AuNPs’ surfaces.

**Scheme 1.**
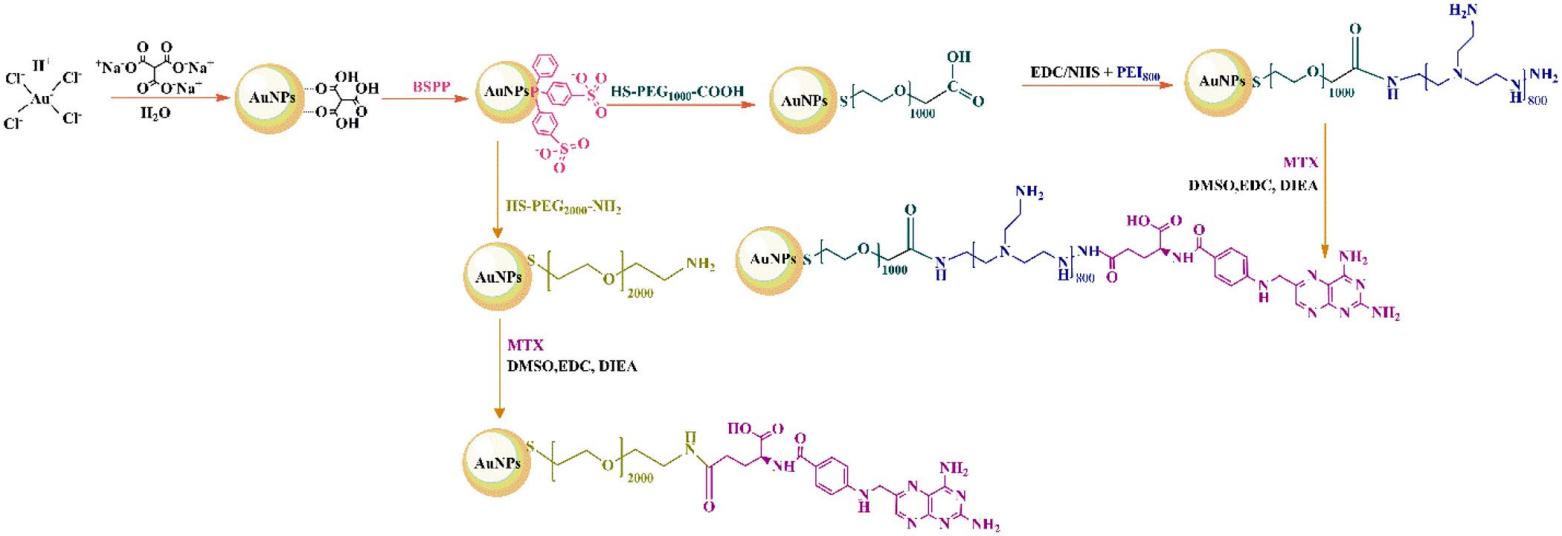
Synthetic pathway for the development of multi-shell gold nanoparticles functionalized with MTX. AuNPs: gold nanoparticles; BSPP: bis(p-sulfonatophenyl) phenyl phosphine; EDC: N-ethyl-N′-(3-dimethylaminopropyl) carbodiimide hydrochloride; NHS: N-hydroxy succinimide; PEG: poly(ethylene glycol); PEI_800_: polyethyleneimine of 800 Da; MTX: methotrexate; DMSO: dimethyl sulfoxide; DIEA: N,N-diisopropylethylamine.

The first nano-entity, AuNPs–PEG–NH_2_, was further coupled with MTX using EDC/DIEA chemistry for the activation of the carboxylic group on the drug. This method allows the covalent bonding of MTX through its carboxylic group on the AuNPs–PEG–NH_2_’s surfaces, generating the first MTX-functionalized nano-entity, AuNPs–PEG–MTX. The second nano-entity, AuNPs–PEG–COOH, was gradually functionalized as required by using EDC/NHS chemistry to add well-defined short-branched PEI as the second shell, resulting in AuNPs–PEG–PEI conjugates. The resulting nano-entities were then functionalized with MTX, as previously reported, to produce AuNPs–PEG–PEI–MTX conjugates. The synthetic pathway is depicted in Scheme 1. The nano-entities obtained were physical–chemical characterized using UV–vis spectroscopy, FTIR, DLS, zeta potential, STEM, and XPS methods.

### 3.1. Ultraviolet–visible spectroscopy

Spherical AuNPs typically absorb visible light, but their oscillation frequency can be modified by changing their size, shape, and the dielectric constant of their environment. These modifications can alter their scattering and absorption spectra. Conversely, by measuring the scattering and absorption properties of AuNPs, it is possible to determine their size, shape, and absorption characteristics (Sharifi et al., 2019). The use of PEG-coating is known to improve the surface properties of nanoparticles by increasing steric hindrance among particles and hydrophilicity, leading to a more stable formulation overall (Rahman et al., 2021). Furthermore, by employing carboxylic groups on the repeating structural unit of the coating polymer, the covalent attachment of MTX to the nanoparticle surface can be quantified using UV–vis spectroscopy. This technique offers a simple, rapid, and effective way to investigate the interaction between nanoparticles and the therapeutic agent, providing valuable insights into the design and optimization of nanoparticle-based drug delivery systems. Next, by applying the ‘Beer–Lambert law’ (Tang et al., 2018) (Equation (1)), the determined concentration of AuNPs dispersed within the solution was *C*_M_ = 92.4 nM. Should be mentioned that in Equation (1), the extinction coefficient (*ε_ext_*) is influenced by both the size of the nanoparticle, as determined through STEM, and the specific wavelength at which it displays its maximum absorption peak (Kim et al., 2015):

(1) $$A=\varepsilon_{\text{ext}}\text{BC}$$

where *A* is the absorbance, *B* is the light path length of the cell (1 cm) used in the UV–vis experiments and $\varepsilon_{\text{ext}}=$8 × 10^8^ cm^−1^ M^−1^.

**Figure 1.**
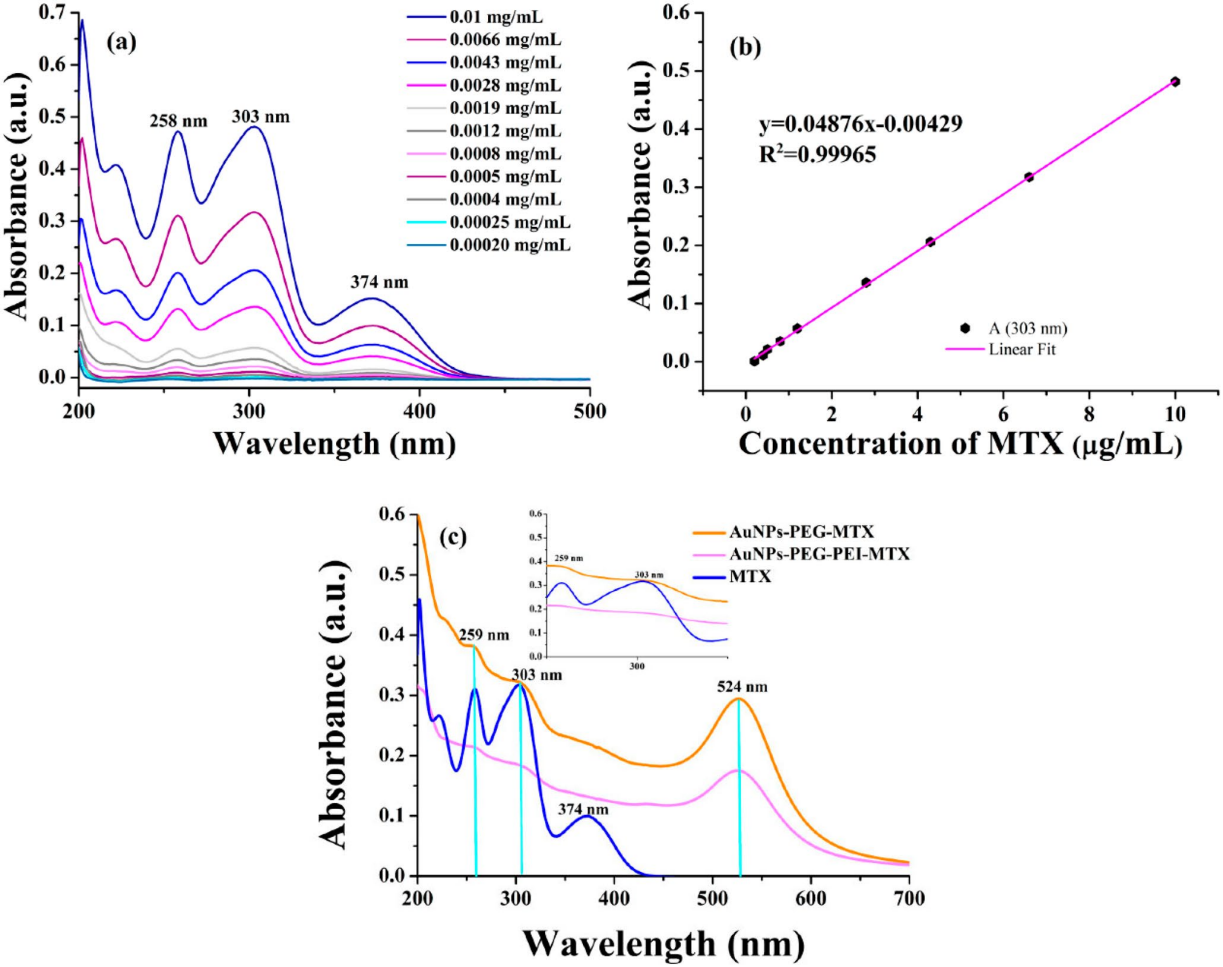
The UV–vis spectra of: (a) methotrexate in water at various concentrations, (b) the calibration curve of methotrexate in water, and (c) AuNPs–PEG–MTX, AuNPs–PEG–PEI–MTX, and MTX.

Free MTX exhibited three characteristic UV–vis absorption peaks at 258 nm, 303 nm, and 374 nm (Figure 1(a)). The presence of two peaks characteristic for MTX at 303 nm and 259 nm on the spectra of functionalized AuNPs (Figure 1(c)) confirmed the binding of MTX on the surface of the polymers, as previously reported (Tran et al., 2013). Using the calibration curve of MTX in water (Figure 1(b)), the calculated MTX concentrations in AuNPs were 0.67 mg MTX/mL in AuNPs–PEG–MTX and 0.385 mg MTX/mL in AuNPs–PEG–PEI–MTX.

### 3.2. Fourier-transform infrared spectroscopy

**Figure 2.**
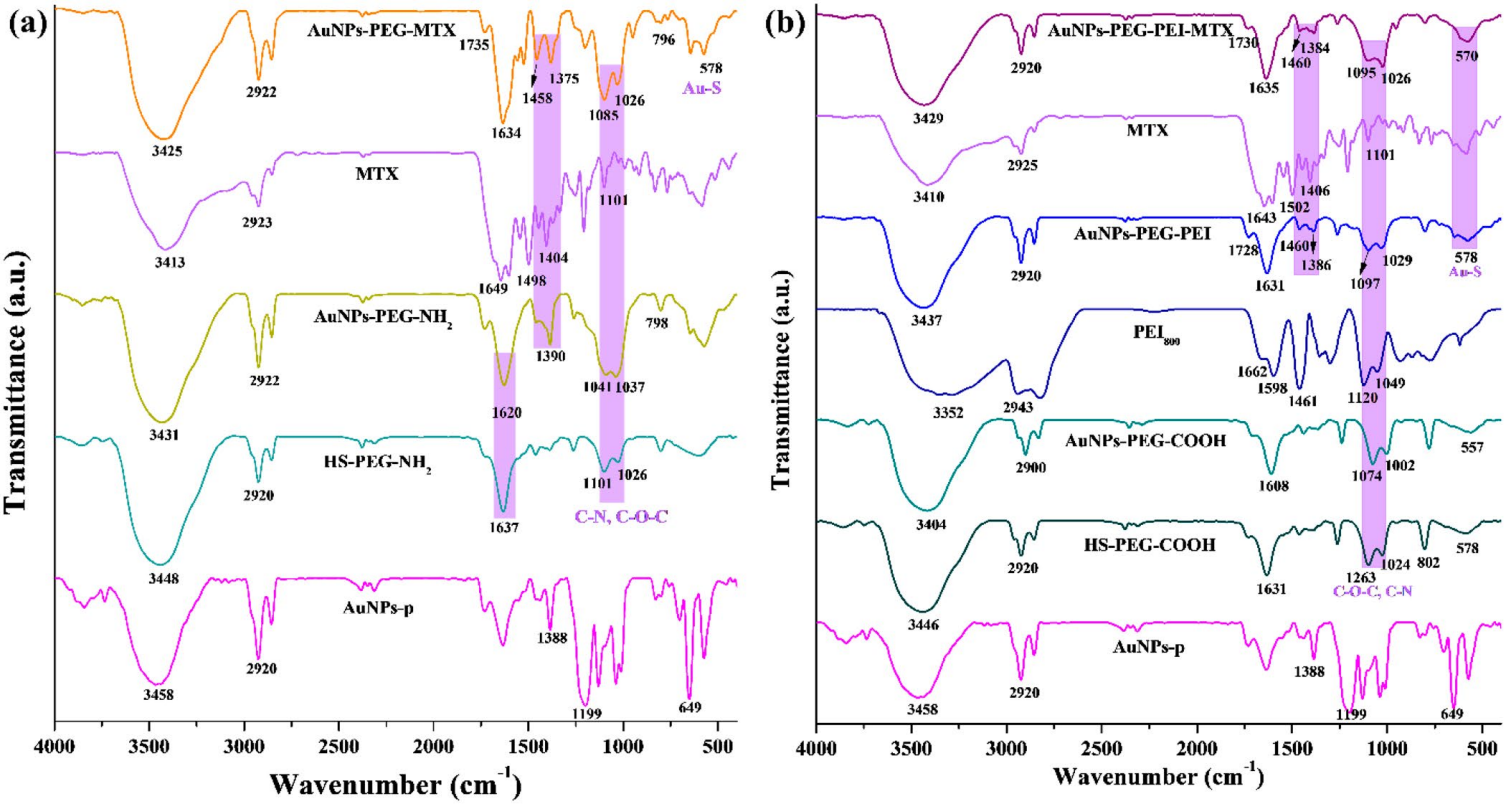
The FTIR spectra of (a) AuNPs–PEG–MTX and each component of the synthesis and (b) AuNPs–PEG–PEI–MTX and each component of the synthesis.

The FTIR spectra of functionalized AuNPs are illustrated in Figure 2(a,b). The FTIR spectrum of AuNPs–PEG–NH_2_ reveals the characteristic peaks of primary aliphatic amines at 3431 cm^−1^, as well as another peak at approximately 1620 cm^−1^ that aligns with the S–C–C bond (Uritu et al., 2024), as demonstrated by FTIR spectrum of HS–PEG–NH_2_ polymer (Figure 2(a)) and the characteristic bands of C–O–C and C–N at around 1100 cm^−1^. In the case of AuNPs–PEG–PEI conjugates, FTIR spectrum (Figure 2(b)) confirms its expected structure by detecting absorption bands typical of primary and secondary amines at 1662 cm^−1^, 1598 cm^−1^, and 1631 cm^−1^, respectively (Uritu et al., 2024), a carboxylic characteristic band at 1728 cm^−1^, and the other characteristic bands attributed to etheric and C–N bonds. FTIR spectra of AuNPs–PEG–MTX and AuNPs–PEG–PEI–MTX contain all characteristic overlapping bands according to their structures (peaks between 1300 and 1500 cm^−1^ which are assigned to the successful binding of MTX; Wang et al., 2016), pointing out that the split of C═O stretching vibration from free MTX is found in a broad peak at around 1635 cm^−1^, additionally confirming the binding of MTX to the AuNPs–PEG–NH_2_ and AuNPs–PEG–PEI conjugates (Fuliaş et al., 2014). Overall, the FTIR spectra of the modified AuNPs exhibited bands ranging from 470 to 800 cm^−1^, indicating the formation of Au–S bonds. At approximately 2900 cm^−1^, the stretching vibration of the C–H bond was observed, while the remaining peak at 3400–3300 cm^−1^ was attributed to the stretching vibration of the NH_2_, C═O, and O–H groups.

### 3.3. Dynamic light scattering and zeta potential

**Figure 3.**
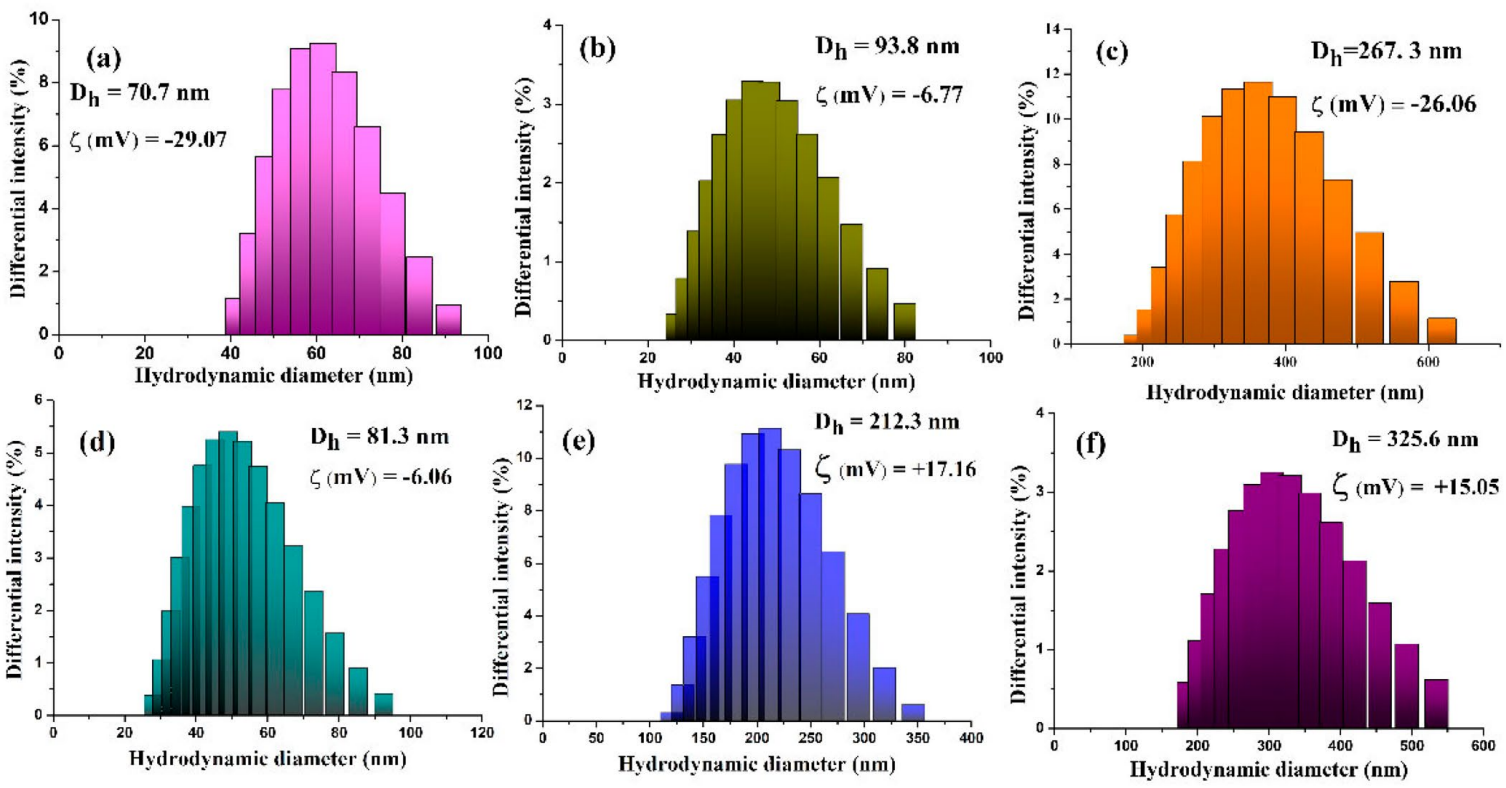
The hydrodynamic diameter (*D*_h_) and zeta potential (*ζ*) of the investigated nanoparticles: (a) AuNPs-p, (b) AuNPs–PEG–NH_2_, (c) AuNPs–PEG–MTX, (d) AuNPs–PEG–COOH, (e) AuNPs–PEG–PEI, and (f) AuNPs–PEG–PEI–MTX.

The hydrodynamic diameter of AuNPs-p presented a dimension of 70 nm and a zeta potential of −29.07 mV, which confirmed the stability of nanoparticles (Figure 3(a)). The binding of HS-PEG-NH_2_ to the AuNPs-p surface is supported by the hydrodynamic diameter value that undergoes from 70 to 93.8 nm while the zeta potential value is from −29.06 mV to +4.51 mV. This last finding is explained by the distribution of amine groups on the modified nanoparticles’ surface, which aims to raise the zeta potential values toward positive values. Going further with the functionalization of AuNPs–PEG–NH_2_ with the MTX drug, the hydrodynamic diameter and zeta potential values varied from 93 to 267 nm and from +4.51 to −26.06 mV, respectively. The decrease in zeta potential can be attributed to the interaction of PEG length with different amino groups (Vasiliu et al., 2021). In our case, the interaction between MTX and PEGylated nanoparticles is influenced by the drug’s structure, which has pKa values of 3.8, 4.8, and 5.6 (Zhao et al., 2020) resulting in a more negative zeta potential value.

The hydrodynamic diameter of AuNPs–PEG–COOH conjugates (Figure 3(d)) is approximately 81 nm, which is smaller than that of the AuNPs–PEG–NH_2_ conjugate. This difference can be attributed to the lower molecular weight of the SH-PEG_1000_-COOH polymer employed in the functionalization of AuNPs, compared to its homolog used for the functionalization of the AuNPs-PEG_2000_-NH_2_ conjugate. On the other hand, the zeta potential value has a negative trend (−16.37 mV) due to the presence of carboxylic groups on the surface of the conjugates. The hydrodynamic diameter, determined by PEI coupling, rises when the PEI polymer is coupled to the surface of AuNPs–PEG–COOH conjugates (Figure 3(e)), and the zeta potential value changes from a negative value to a positive value (from −16.37 to +17.16 mV). The coupling of MTX to the AuNPs–PEG–PEI nanosystem (Figure 3(f)) resulted in an observed increase in the hydrodynamic diameter and a reduction in the zeta potential value (+15.05 mV), which aligns with the observed trend seen in the AuNPs–PEG–MTX nanosystem.

### 3.4. Scanning transmission electron microscopy

**Figure 4.**
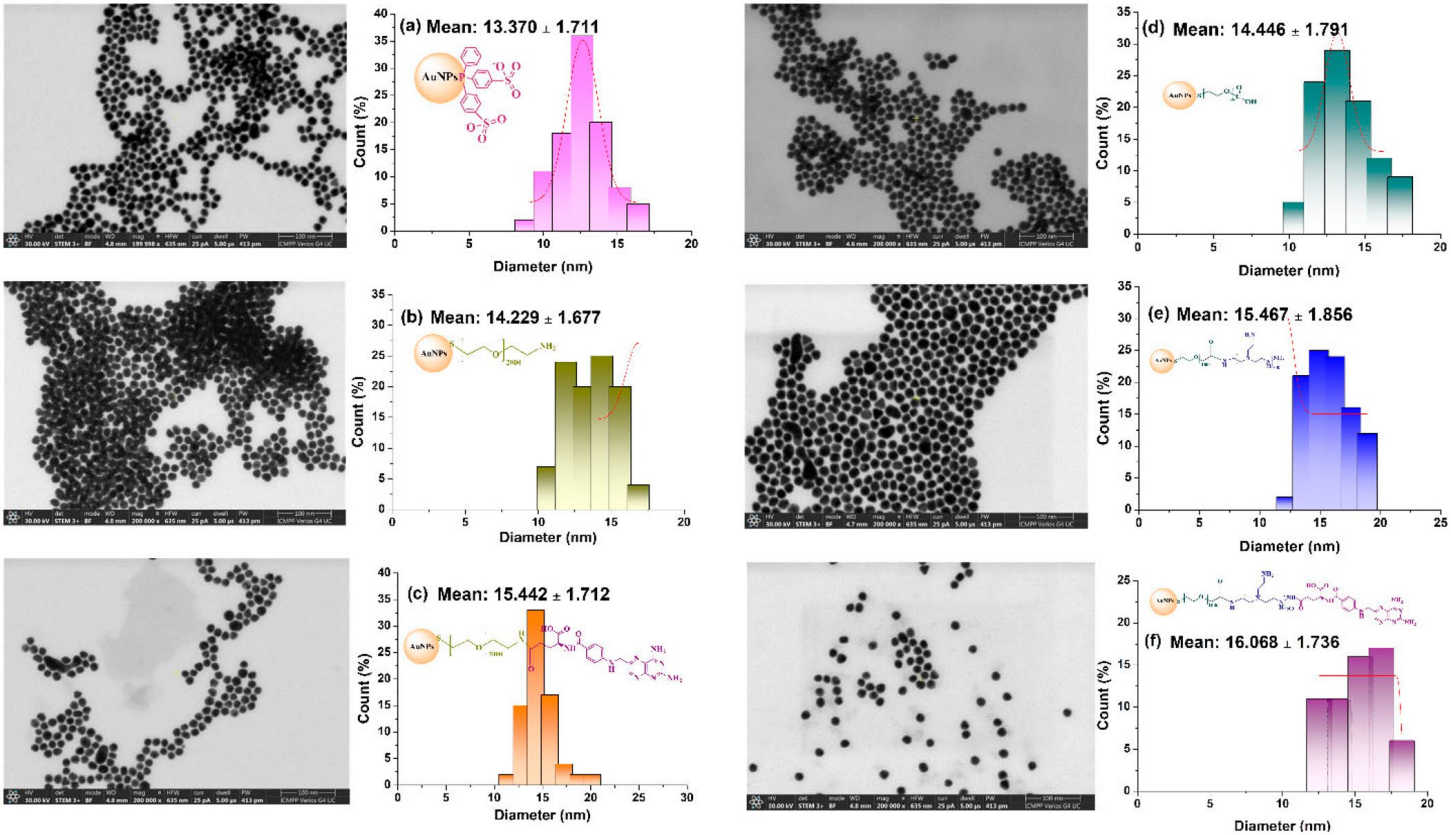
Scanning transmission electron microscopy images and the histograms of the size distribution of the investigated nanoparticles*:* (a) AuNPs-p, (b) AuNPs–PEG–NH_2_, (c) AuNPs–PEG–MTX, (d) AuNPs–PEG–COOH, (e) AuNPs–PEG–PEI, and (f) AuNPs–PEG–PEI–MTX.

The formation of spherical morphology of uncovered and covered AuNPs is further supported by STEM imaging (Figure 4). The nanoparticles exhibit a low polydispersity and have mean diameters of 13 ± 1.7 (AuNPs-p), 14 ± 1.6 (AuNPs–PEG–NH_2_), 15 ± 1.7 (AuNPs–PEG–MTX), 14 ± 1.7 (AuNPs–PEG–COOH), 15 ± 1.8 (AuNPs–PEG–PEI), and 16 ± 1.7 (AuNPs–PEG–PEI–MTX). As anticipated, subsequent to the functionalization of the surface, the dimensions of the nanoparticles exhibit a progressive enlargement at each successive step. The STEM investigation demonstrated that the diameters of nanoparticles were smaller in comparison to the data obtained by DLS. The disparities seen between DLS and STEM may be attributed to the interactions between the aqueous medium and particle surface layer in DLS measurements, namely the size and charge of the PEG, PEI, and MTX molecules (Craciun et al., 2023).

### 3.5. X-ray photoelectron spectroscopy

**Figure 5.**
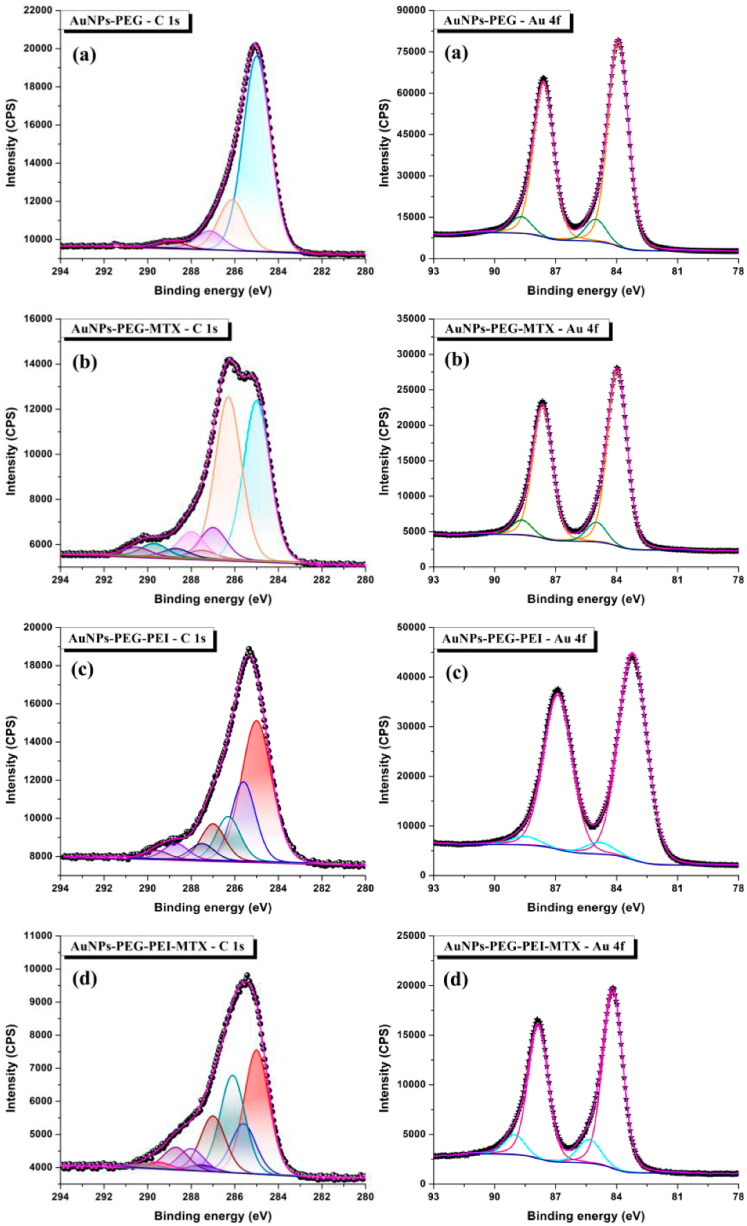
C 1s and Au 4f high-resolution spectra of (a) AuNPs–PEG–NH_2_, (b) AuNPs–PEG–MTX, (c) AuNPs–PEG–PEI, and (d) AuNPs–PEG–PEI–MTX.

The surface composition of the functionalized AuNPs was studied using the XPS method. Figure 5 illustrates the high-resolution spectra of C 1s and Au 4f for the different AuNPs. The C 1s high-resolution spectra of AuNPs–PEG–NH_2_ exhibit four distinct peaks at 285, 286.1, 287.2, and 288.9 eV. These peaks may be assigned to different carbon species, namely adventitious carbons (C–C or C–H), primary amine (C–NH_2_), ether (C–O–C), and carbon–sulfur bonds arising from the heterobifunctional PEG chain (C–S), respectively (Lupusoru et al., 2020; Thambiraj et al., 2021). The C 1s signal of the AuNPs–PEG–MTX nano-entity was further analyzed, and it was shown to have additional peaks, confirming the functionalization of AuNPs–PEG–NH_2_ with MTX. These peaks, for instance, reflected the existence of the C═N peak originating from the pteridine ring or the formation of an amide bond between the –NH_2_ group and one of the carboxylic units from the glutamic acid component of the MTX. To be more specific, eight individual peaks could be seen in the C 1s signal of the AuNPs–PEG–MTX, with energies located at 285, 286.3, 287, 287.5, 288, 288.7, 289.6, and 290.3 eV, respectively. The peaks that were identified were assigned to several chemical groups, including adventitious carbons (C–C or C–H), primary amine (C–NH_2_), tertiary amine (C–N–C), ether (C–O–C), carbon–nitrogen bond from the pteridine ring (C═N), carbon–sulfur (C–S), amide (O═C–N), and carboxylic (O═C–O) bonds (Lupusoru et al., 2020).

In contrast to the aforementioned two AuNPs, the addition of the branched PEI unit onto the PEGylated AuNP, AuNPs–PEG–PEI, resulted in the emergence of seven distinct peaks in the C 1s spectra located at 285, 285.6, 286.3, 287, 287.5, 288.7, and 289.6 eV. The observed signals may be attributed to the presence of the adventitious carbons (C–C or C–H), secondary amine (C–NH), primary amine (C–NH_2_), tertiary amine (C–N–C), ether (C–O–C), carbon–sulfur (C–S), and amide (O═C–N), respectively. In addition, the high-resolution C 1s spectra of the AuNPs–PEG–PEI–MTX nano-entity exhibited nine distinct peaks positioned at 285, 285.6, 286.1, 287, 287.5, 288, 288.7, 289.5, and 290 eV which can be attributed to the adventitious carbons (C–C or C–H), secondary amine (C–NH), primary amine (C–NH_2_), tertiary amine (C–N–C), ether (C–O–C), carbon–nitrogen bond from the pteridine ring (C═N), carbon–sulfur (C–S), amide (O═C–N), and carboxylic (O═C–O) bonds, respectively (Lupusoru et al., 2020; Thambiraj et al., 2021).

The existence of Au(0) and Au(1) on the surface of all four AuNPs was confirmed through examination of the high-resolution spectra of the Au 4f (Park & Shumaker-Parry, 2014; Lupusoru et al., 2020), as shown in Figure 5. The coordination of Au-carboxylate and Au–S may explain the presence of Au(1) species on the surface of AuNPs (Huo et al., 2008; Park & Shumaker-Parry, 2014).

### 3.6. Antioxidant activity

**Figure 6.**
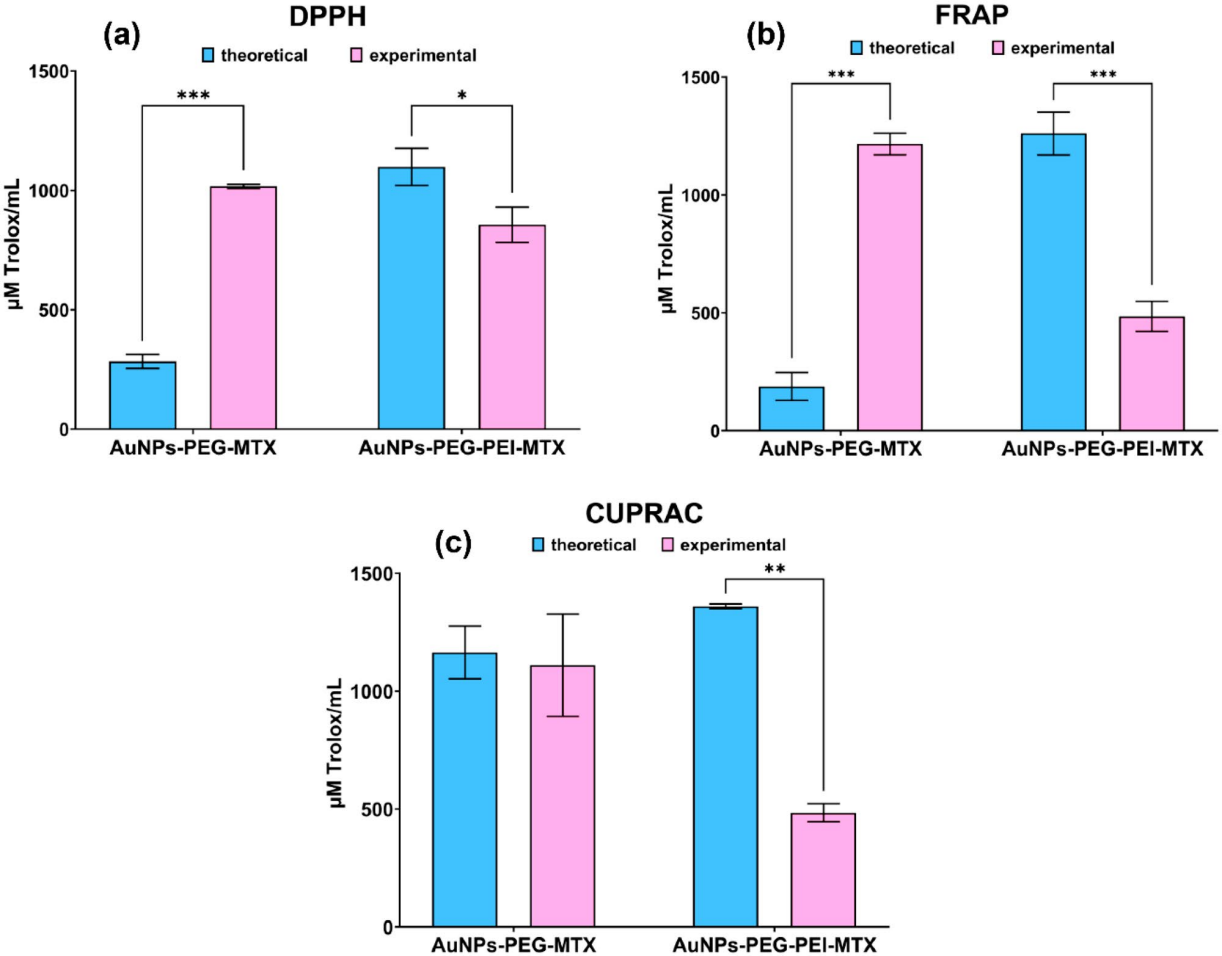
Comparative evaluation of the antioxidant activity of the individual components’ sum (theoretical) and functionalized nanoparticles (experimental): (a) DPPH assay, (b) FRAP assay, and (c) CUPRAC assay.

The antioxidant activity was determined for both AuNPs functionalized with MTX, as well as for the intermediate functionalization products. As depicted in Figure 6, a synergistic effect was evident for AuNPs–PEG–MTX, where the functionalized nanoparticles exhibited a significantly higher result (*p* < .001) than the combined antioxidant activity of individual components in both the DPPH and FRAP assays. In contrast, an interesting antagonistic effect was observed for AuNPs–PEG–PEI–MTX, where the combined antioxidant activity of the individual components surpassed that of the functionalized nanoparticles, as demonstrated by the DPPH (*p* < .05), FRAP (*p* < .001), and CUPRAC (*p* < .01) assays. These results indicate that the combination of components in AuNPs–PEG–MTX and AuNPs–PEG–PEI–MTX led to distinct effects on antioxidant activity, with a synergistic effect in the former, and an antagonistic effect in the latter (Flieger et al., 2021; Uduwana et al., 2023). The findings highlight the importance of understanding the interactions between components in nanoparticles’ functionalization for their potential applications as antioxidants.

**Figure 7.**
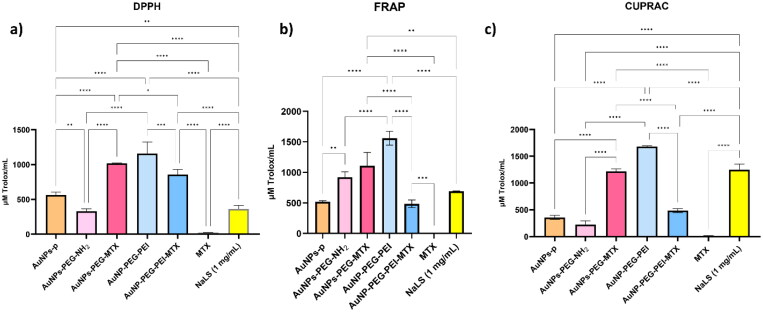
Antioxidant activity of intermediate products and functionalized nanoparticles: (a) DPPH assay, (b) FRAP assay, and (c) CUPRAC assay.

From Figure 7, it is evident that AuNPs–PEG–MTX exhibits significantly higher antioxidant activity compared to AuNPs–PEG–NH_2_ (*p* < .0001 for DPPH and CUPRAC assays) and free MTX (at the same concentration as found in nanoparticles, *p* < .0001 for all three assays). These findings emphasize the enhanced antioxidant potential achieved through MTX loading onto AuNPs–PEG–NH_2_, outperforming both the unfunctionalized PEGylated AuNPs and free MTX alone.

In contrast, AuNPs–PEG–PEI–MTX has significantly lower antioxidant activity compared to its precursor, as evidenced by the DPPH (*p* < .001), FRAP (*p* < .0001), and CUPRAC (*p* < .0001) assays. The potent antioxidant activity observed in the AuNPs–PEG–PEI intermediate product can be attributed to the presence of free hydroxyl groups from PEG. Conversely, the decrease in antioxidant activity upon functionalization with MTX can be explained by the interaction of the hydroxyl groups with the glutamic group in the MTX structure. These findings underscore the significance of the intermediate product, AuNPs–PEG–PEI, in conferring robust antioxidant properties, and shed light on the influence of MTX functionalization on the overall antioxidant activity of the nanoparticles. Understanding such interactions is crucial in tailoring the antioxidant behavior of functionalized nanoparticles for potential applications in various fields.

Remarkably, in both cases, the antioxidant activity of the functionalized nanoparticles is considerably higher than that of free MTX at the same concentration as that embedded in the nanoparticles, suggesting the advantageous role of MTX functionalization in promoting antioxidant capabilities. Furthermore, upon comparing AuNPs–PEG–MTX with AuNPs–PEG–PEI–MTX, it can be concluded that AuNPs–PEG–MTX exhibits the highest antioxidant activity, as evidenced by the results obtained from the DPPH (*p* < .05), FRAP (*p* < .0001), and CUPRAC (*p* < .0001) assays. The DPPH method revealed that the AuNPs-p (*p* < .01), AuNPs–PEG–MTX (*p* < .0001), AuNPs–PEG–PEI (*p* < .0001), and AuNPs–PEG–PEI–MTX (*p* < .0001) had significantly greater antioxidant activity than the NaLS standard (Figure 7(a)). In the FRAP method, only the AuNPs–PEG–MTX (*p* < .01) and AuNPs–PEG–PEI (*p* < .0001) variants showed significantly higher antioxidant activity compared to the NaLS standard (Figure 7(b)). In the CUPRAC method, only AuNPs–PEG–PEI (*p* < .0001) had higher activity compared to NaLS (Figure 7(c)). The high antioxidant activity of the NaLS solution can be attributed to the lignin’s large number of phenolic monomers. The phenolic monomers convert the DPPH radical and reduce Cu(II) to Cu(I) and Fe(III) to Fe(II), through an electron-proton transfer mechanism (Novo & Edgar, 2024). MTX is known for its anti-cancer, anti-inflammatory, immunosuppressive, and apoptotic effects, which are partly due to its ability to increase ROS levels in both healthy and cancerous cells. This mechanism highlights MTX's absence of antioxidant properties, leading to a compromised antioxidant defense system and heightened vulnerability to ROS-induced damage (Safaei et al., 2018). Contrarily, the nano-carriers used for MTX delivery exhibit significant antioxidant capabilities, attributed to the presence of PEG (Zhang et al., 2022). This combination potentially mitigates the harmful effects of MTX, offering a more balanced therapeutic approach (Sener et al., 2006; Vardi et al., 2009; Mahoutforoush et al., 2023).

### 3.7. Cytocompatibility of the functionalized AuNPs on normal versus tumoral skin cells

**Figure 8.**
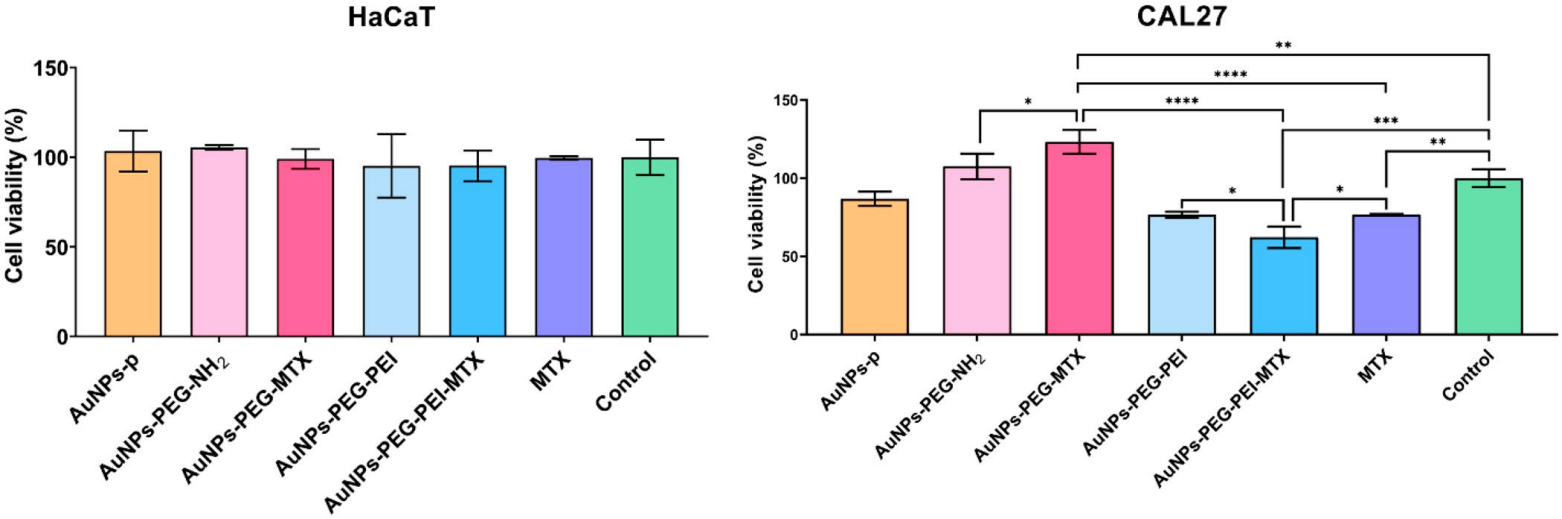
Evaluation of the biocompatibility of gold nanoparticles functionalized with MTX by the MTT assay under standard cultivation conditions of the HaCaT human keratinocyte cell line (left) and CAL27 squamous cell carcinoma cell line (right).

Figure 8 indicates that the HaCaT cellular viability was comparable to that of the control group when using the MTT assay. There were no statistically significant differences observed, both for the intermediate products and MTX, as well as for the nanoparticles functionalized with MTX when compared to the control. Different results have been obtained when using the CAL27 squamous cell carcinoma cell line in this case. The AuNPs–PEG–PEI–MTX proved the best ability to decrease the viability of the tumoral cells, followed by MTX and AuNPs–PEG–PEI.

Overall, these results suggest that the tested samples, including the functionalized nanoparticles with MTX, did not cause a notable decrease in cell viability compared to the control group.

### 3.8. Cytotoxic effects of functionalized AuNPs on normal versus tumoral skin cells

**Figure 9.**
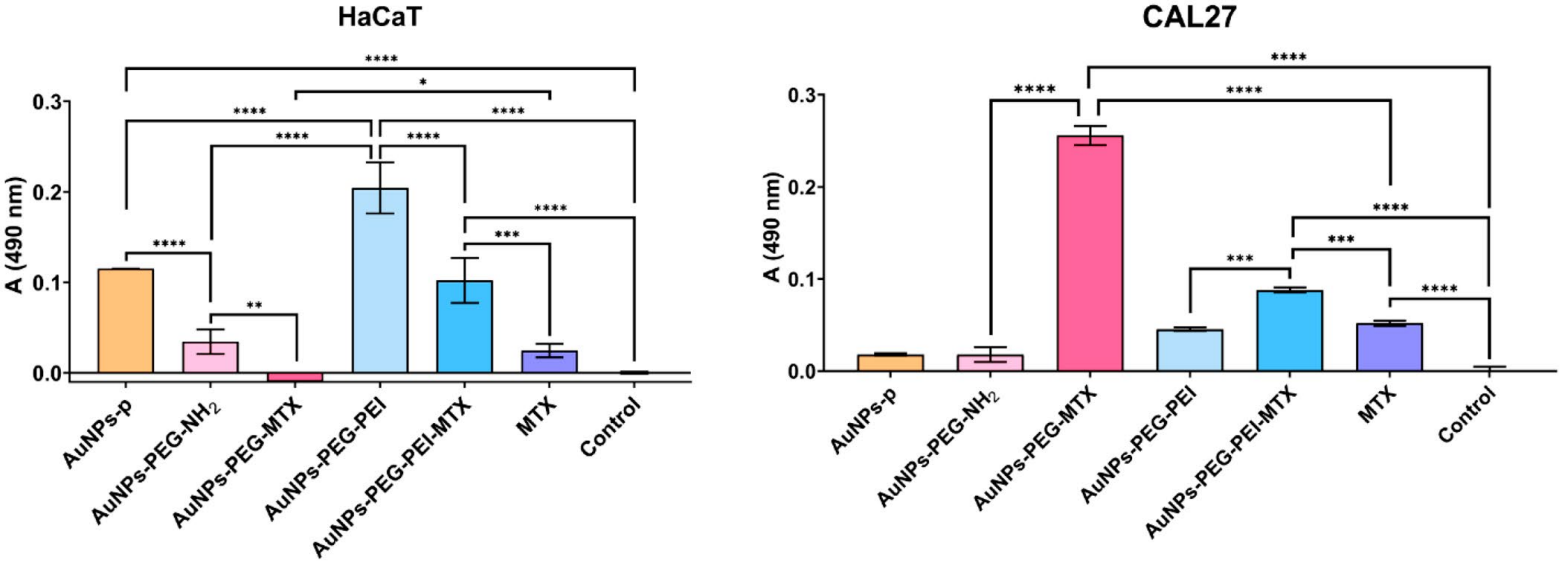
Evaluation of nanoparticles’ cytotoxicity by the LDH assay under standard cultivation conditions of HaCat (left) and CAL27 squamous cell carcinoma cell lines (right).

The results from the LDH assay on HaCaT and CAL27 cells consistently demonstrated a more pronounced cytotoxic effect of the functionalized AuNPs on the tumoral in comparison with normal cells, especially in the case of AuNPs–PEG–MTX (Figure 9). The cytotoxic effect of MTX-functionalized AuNPs on CAL27 cells was significantly higher than the same concentration of free MTX (*p* < .001). It is noteworthy that even non-functionalized nanoparticles (those without MTX) exhibited a statistically significant cytotoxic effect on the tested tumoral cells (*p* < .05 for AuNPs-p and AuNPs–PEG–NH_2_, and *p* < .0001 for AuNPs–PEG–PEI).

The findings of this study highlight the cytotoxic potential of multi-shell AuNPs in enhancing the anti-tumor properties of MTX. Moreover, the documented cytotoxicity of AuNPs in the absence of MTX emphasizes the need to comprehensively assess the inherent characteristics of nanomaterials in order to have a complete understanding of their biological interactions.

**Figure 10.**
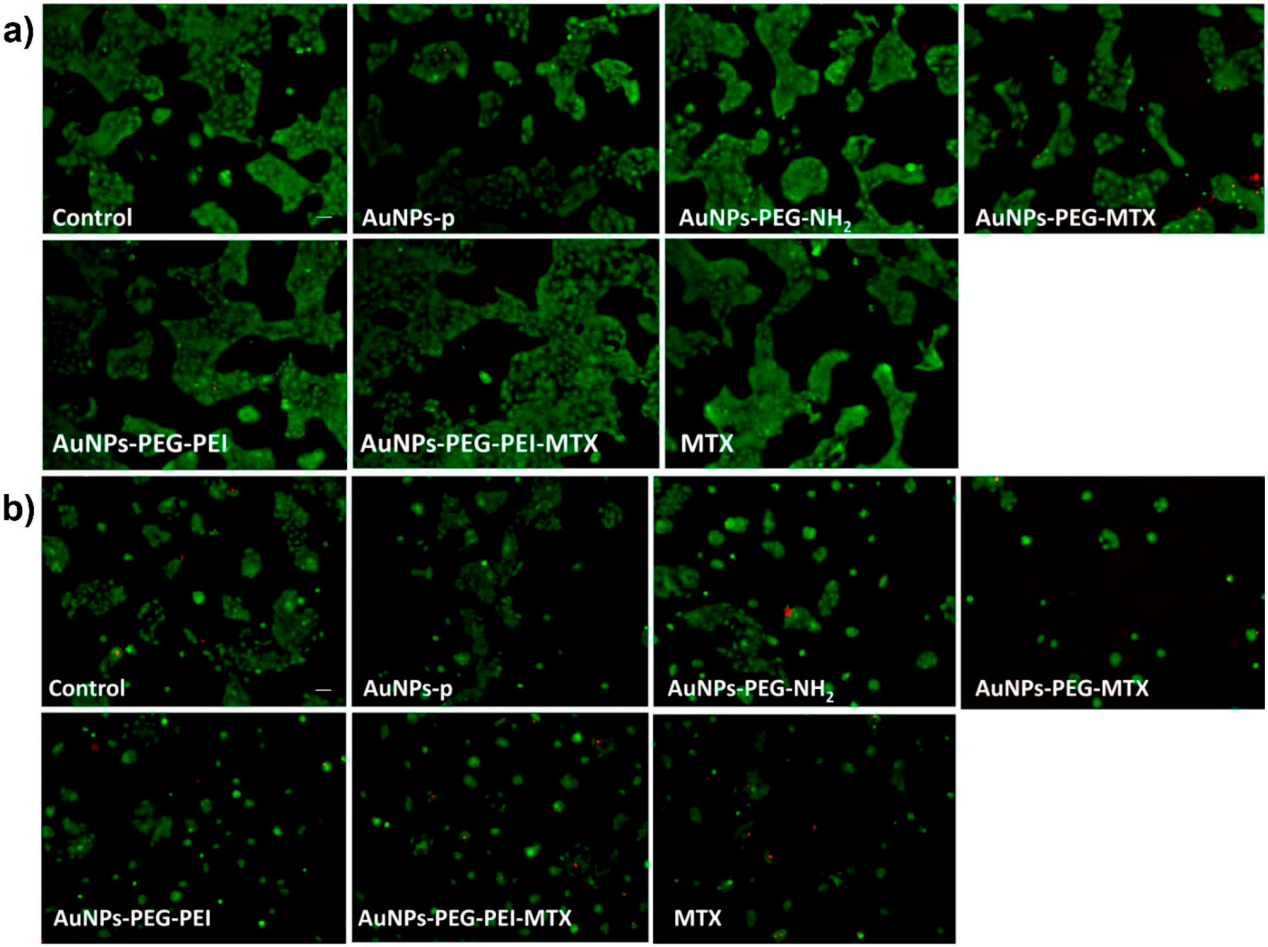
Cell viability measured by Live/Dead assay. (a) HaCat (normal) cells cultivated for 24 hours in the presence of functionalized AuNPs; (b) tumoral (CAL27) cells cultivated for 24 hours in the presence of functionalized AuNPs; scale bar – 100 µm.

The Live/Dead assay corroborates the findings from biochemical tests, as demonstrated in Figure 10. Specifically, HaCat cells displayed a markedly higher ratio of live (green) to dead (red) cells, indicating cell viability in Figure 10(a). Conversely, tumor cells presented reduced viability in the presence of AuNPs, as shown in Figure 10(b).

While AuNPs are generally considered nontoxic, the literature presents mixed findings regarding their potential adverse effects (Uboldi et al., 2009; Zhang et al., 2012; Bhamidipati & Fabris, 2017; Vetten & Gulumian, 2019). The cytotoxicity of AuNPs can vary significantly depending on their shape, surface chemistry, size, the type of cells involved, and the mode of application (Bhamidipati & Fabris, 2017; Vales et al., 2020; Sani et al., 2021). Studies have shown that when applied to *in vitro* systems, the toxic effects of AuNPs can differ based on the cell type used (Coradeghini et al., 2013; Lillo et al., 2020). For instance, HL-60 cells exhibited greater susceptibility to cytotoxic effects compared to HepG2 cells in studies focused on AuNPs-induced cytotoxicity and oxidative stress (Aljarba et al., 2022). The generation of intracellular ROS has been identified as a mechanism through which AuNPs exert cytotoxic effects, with some research indicating that smaller nanoparticles may lead to increased ROS production (Sen et al., 2020). However, not all studies observed changes in intracellular ROS levels, such as in Caco-2 cells (Aueviriyavit et al., 2014). This discrepancy may be attributed to the antioxidant effects of coatings like PEG or PEG-PEI, which can reduce oxidative stress and impart a selective cytotoxicity profile to the nanoparticles (Juarez-Moreno et al., 2015; Jwameer et al., 2023).

## 4. Conclusions

The physicochemical properties of functionalized AuNPs with MTX lead to the formation of stable nano-systems organized in large clusters with a close-packed arrangement. This self-assembling property is of great importance for drug delivery purposes as it affects the size, functionality, and morphology of the drug-loaded nanoparticles.

The functionalized nanoparticles demonstrated enhanced antioxidant activity compared to that of free MTX, their components, and intermediate products. Additionally, the AuNPs with MTX exhibited a more pronounced cytotoxic effect on tumor cells, highlighting their potential as a candidate for improving cancer therapy. Furthermore, the study revealed that even the nanoparticles without MTX exerted a statistically significant cytotoxic effect on the tested tumor cells, emphasizing the inherent anti-cancer effect of AuNPs. However, it is essential to note that the normal cell viability remained similar to that of the control group for all tested nanoparticles, including the MTX-functionalized nanoparticles, indicating minimal adverse effects on overall cell health. These findings suggest that AuNPs functionalized with MTX hold promise for various biomedical applications, such as targeted cancer therapy and antioxidant-based treatments. However, further research is needed to optimize their performance, ensure safety, and explore their potential in clinical settings.

Understanding how these systems behave can aid in enhancing drug efficacy and provide insights into the pharmacodynamics and pharmacokinetic properties. This can open up new possibilities for therapy and drug delivery systems, thus expanding our understanding of the physicochemical mechanisms involved.

## Data Availability

The data that support the findings of this study are available from the corresponding authors [N.S. and M.P.], upon reasonable request.
